# Gallstone ileus in a young patient complicated by double biliary-enteric fistula: A rare case report

**DOI:** 10.1016/j.ijscr.2025.111946

**Published:** 2025-09-16

**Authors:** Mohamed Zayati, Mohamed Ali Chaouch, Ibtissem Korbi, Midani Touati, Bassem Bouzouita, Faouzi Noomen

**Affiliations:** a Department of Visceral and Digestive Surgery, Monastir University Hospital, Tunisia; b Department of Anesthesiology, Monastir University Hospital, Tunisia

**Keywords:** Gallstone ileus, Biliary-enteric fistula, Cholecysto-duodenal fistula, Cholecysto-colic fistula, Small bowel obstruction, Case report

## Abstract

**Background:**

Gallstone ileus is an uncommon complication of chronic cholecystitis, characterized by mechanical small bowel obstruction due to gallstone migration through a biliodigestive fistula. The occurrence of double biliary-enteric fistulas (e.g., cholecysto-duodenal and cholecysto-colic) is infrequent and presents additional diagnostic and surgical challenges.

**Case presentation:**

A 41-year-old woman with no significant past medical history presented with progressive abdominal pain, distension, and cessation of bowel movements. Laboratory findings indicated a severe inflammatory response (CRP: 312 mg/L; leukocytosis: 13,000/mm^3^). CT imaging revealed jejuno-ileal distention with a calcified intraluminal mass suggestive of gallstone ileus and evidence of a cholecysto-duodenal fistula. Emergency laparotomy confirmed a large obstructing gallstone 30 cm from the ileocecal valve. Surgical exploration revealed two fistulas: cholecysto-duodenal and cholecysto-colic. The stone was removed via enterotomy; both fistulas were sutured, and a partial cholecystectomy was performed. The postoperative course was uneventful.

**Discussion:**

Gallstone ileus typically affects older individuals, and its occurrence in a young patient is unusual. The presence of a double fistula is a rare complication that complicates surgical management. Early diagnosis via imaging and timely intervention are critical. In this case, a one-stage procedure involving enterotomy, fistula closure, and partial cholecystectomy was successfully performed.

**Conclusion:**

This case underscores the diagnostic and surgical complexity of gallstone ileus, especially when associated with double biliary-enteric fistulas. It emphasizes the importance of individualized surgical planning and the utility of CT imaging in diagnosis.

## Highlights

• The case involves a 41-year-old woman, which is unusual because gallstone ileus typically affects older individuals (usually over 65). Even more exceptional is the presence of **two biliary-enteric fistulas** (cholecysto-duodenal and cholecysto-colic), a combination that significantly complicates diagnosis and surgical intervention.
• CT imaging was crucial in diagnosing gallstone ileus and suggesting a biliodigestive fistula. During emergency laparotomy, the obstructing gallstone was found 30 cm from the ileocecal valve and removed via enterotomy. Both fistulas were sutured, and a partial cholecystectomy was performed in a **one-stage surgical procedure**, a challenging but successful approach given the patient's condition.
• The case underscores the importance of **early imaging and individualized surgical planning.** It illustrates how a one-stage operation can be effectively executed in complex presentations like double fistulas, avoiding the need for staged surgeries that might carry higher risks of postoperative complications.

## 1 Introduction

Gallstone ileus is a very rare and delayed complication that occurs in patients with a history of gallstone disease complicated by subacute or chronic cholecystitis [1]. It involves a mechanical small bowel obstruction caused by the impaction of a large gallstone (> 2.5 cm) that has perforated the wall of the gallbladder (in the fundus or body) and created a biliodigestive fistula. Also, there is a double fistula connecting the gallbladder with the duodenum and the colon. This is a very rare complication that will be the focus of this report. The objective of this case report is to present a rare and complex case of **gallstone ileus** in a **young female patient**, which was **complicated by a double biliary-enteric fistula**, a highly unusual occurrence [2]. This case report, written according to SCARE guidelines [3], aims to highlight the diagnostic challenges, surgical management decisions, and clinical significance of encountering both a cholecysto-duodenal and cholecysto-colic fistula in the same patient.

## 2 Case presentation

**Fig. 1:**
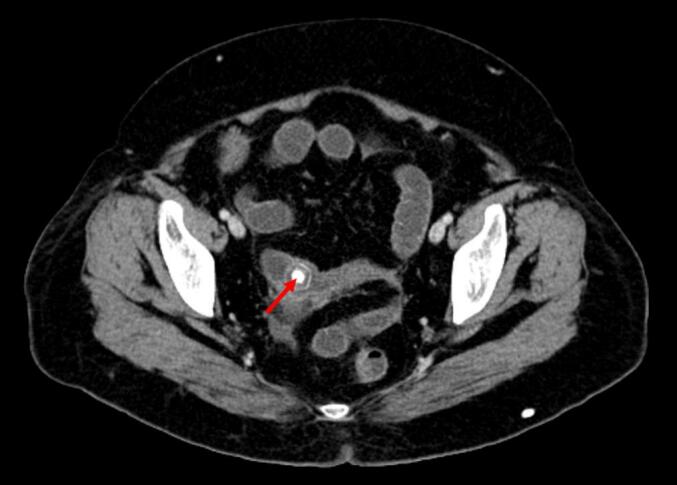
Axial abdominal CT scan view showing the stone in the intraluminal of the small bowel (red arrow). (For interpretation of the references to colour in this figure legend, the reader is referred to the web version of this article.)

**Fig. 2:**
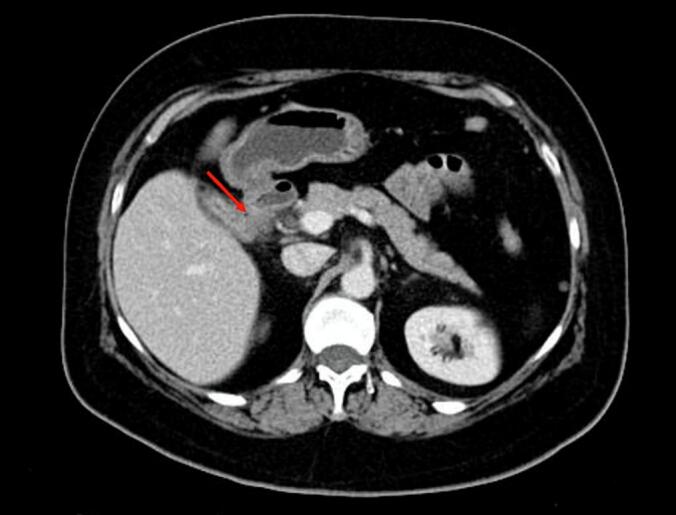
Axial abdominal CT scan view suggesting the cholecysto-duodenal fistula (red arrow). (For interpretation of the references to colour in this figure legend, the reader is referred to the web version of this article.)

**Fig. 3:**
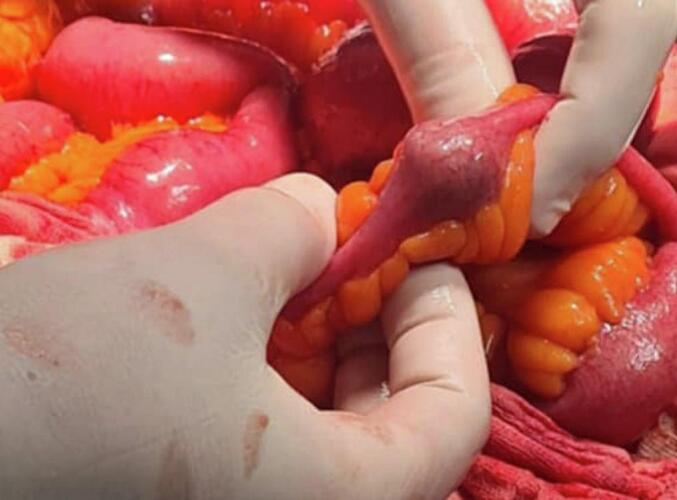
Intraoperative view showing the presence of the gallbladder stone in the small bowel.

**Fig. 4:**
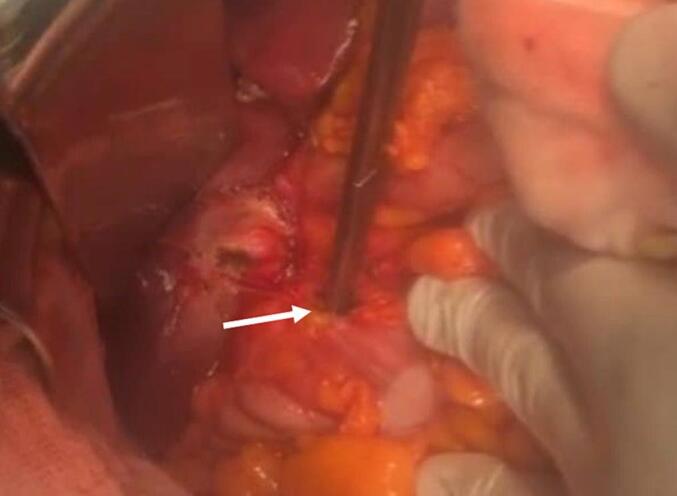
Intraoperative view showing colonic fistula (white arrow).

**Fig. 5:**
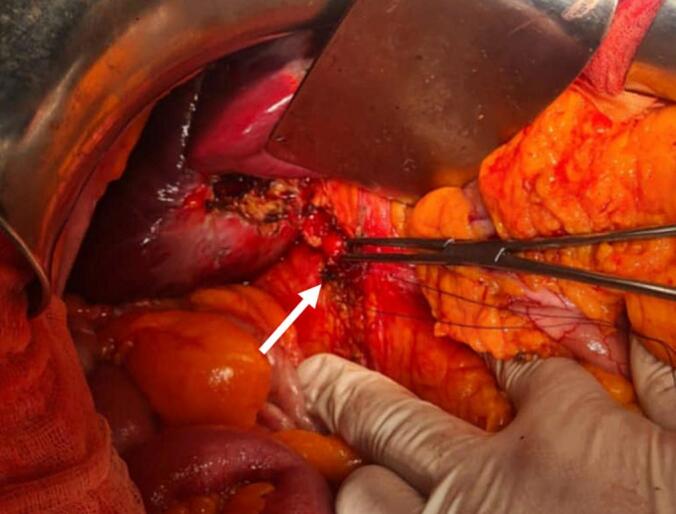
Intraoperative view showing duodenal fistula (white arrow).

A 41-year-old woman patient with no significant past medical history presented with colicky pain in the right upper quadrant that developed over one year. Recently, her condition worsened with the onset of diffuse abdominal pain, abdominal distention, and cessation of both stool and gas passage. Laboratory tests revealed a biological inflammatory syndrome with a C-reactive protein (CRP) level of 312 mg/L and leukocytosis of 13,000 / mm3, without abnormalities of liver function. Given the presence of an occlusive syndrome associated with inflammatory markers, an abdominal CT scan was performed. It showed jejuno-ileal distention (37 mm in diameter) upstream of a transition point involving a distended loop and a collapsed loop in the pelvis, caused by an intraluminal, rounded, calcified structure measuring 18 mm, consistent with a gallstone (Fig. 1). There was also fat stranding in the gallbladder fossa, a collapsed gallbladder with submucosal edema, and suspicion of a cholecysto-duodenal fistulous tract (Fig. 2). An emergency surgical intervention was indicated. A midline laparotomy was performed, revealing distension of the small intestine upstream of a gallstone located 30 cm from the ileocecal valve (Fig. 3). The stone was extracted through an enterotomy. Intraoperatively, two fistulas were identified: one between a scleroatrophic gallbladder and the right colic angle (Fig. 4), and another between the gallbladder and the duodenum (Fig. 5). Both were sutured. A partial cholecystectomy was performed due to technical difficulty in performing a total cholecystectomy associated to an external drainage. The postoperative course was uneventful.

## 3 Discussion

Gallstone ileus is a mechanical small intestine caused by the impaction of a large gallstone (> 2.5 cm) that has perforated the wall of the gallbladder (at the fundus or body) and created a biliodigestive fistula. The clinical presentation varies depending on the location of the obstruction. When the gallstone is impacted in the duodenum, it results in Bouveret syndrome in 3 % of cases. It can also lodge in the ileum in 90 % of cases, causing small bowel obstruction. When the obstruction occurs at the colonic level in 8 % of cases, it leads to low intestinal obstruction [1,2]. This condition predominantly affects female patients. In individuals over 65 years of age, it develops in 0.3 to 0.5 % of those with gallstone disease [4,5]. Pathophysiologically, repeated bouts of lithiasic cholecystitis lead to perivesicular inflammation, leading to the formation of a cholecysto-digestive fistula and migration of gallstones into the gastrointestinal tract [4]. Although gallstone ileus is exceptionally rare in young patients, previous reports have documented similar cases in even younger individuals [6]; however, our case is distinguished by the presence of a double fistula involving both the duodenum and colon, a combination rarely described in the literature. The clinical presentation is characterized by an obstructive syndrome, often preceded by a history of biliary colic. The diagnosis is based on imaging, with a CT scan being the routine diagnostic test. Most often, visualization of a cholecysto-duodenal fistula and precise delineation of the transition zone are enabled, most often in the ileum [6,7]. Double fistula is an exceptional complication of this condition. In the case we reported, we found both a cysto-duodenal fistula and a cysto-colic fistula, which were managed by suturing the colonic and duodenal perforations. Early treatment is crucial. Doctors typically consider two surgical options: removing the intestinal stone alone or removing the stone in conjunction with repairing the fistula of the bile duct and gallbladder removal, which can be performed in one or two separate procedures [7,8]. In a single surgical approach, the management of gallstone ileus through enterotomy associated with concomitant cholecystectomy is frequently complicated by technical difficulties, particularly during gallbladder dissection and closure of associated colonic or duodenal perforations. In such cases, if the patient's clinical condition does not permit prolonging the surgical procedure or if there is a major intraoperative challenge, one may opt to address only biliary obstruction without treating the fistula during the initial operation. However, delaying fistula repair to a second surgical stage may increase postoperative morbidity [9,10].

## 4 Conclusions

Gallstone ileus is a rare condition secondary to chronic cholecystitis. Multiple inflammatory mechanisms can underline this clinical presentation, with several potential sites of stone impaction. A double fistula represents an exceptional complication of this condition, which we encountered in this clinical case.

## Ethical approval

Ethical approval is exempt/waived at our institution, Monastir University Hospital, for all the case reports.

## Funding

None.

## Author contribution

Hafedh Daly, Faiez Boughanmi, Mohamed Ali Chaouch, Midani Touati, Fethi Jebali, and Bahri Mahjoub participated in the manuscript and validated the final version of the manuscript

## Guarantor

Mohamed Ali Chaouch.

## Research registration number

Not applicable.

## Consent

Written informed consent was obtained from the patient for publication and any accompanying images. A copy of the written consent is available for review by the Editor-in-Chief of this journal on request.

## Conflict of interest statement

No conflict of interest to disclose
